# Surgical management of frontoethmoidal encephalocele in a 4-month-old infant: An Ethiopian perspective and case report

**DOI:** 10.1016/j.ijscr.2024.110254

**Published:** 2024-09-07

**Authors:** Anteneh Meaza Dawit, Getaw Alamne Gebeyehu, Salahadin Bedru Mohammed

**Affiliations:** Addis Ababa University, Department of Plastic & Reconstructive Surgery, Addis Ababa, Ethiopia

**Keywords:** Frontoethmoidal encephalocele, Bone graft, Multidisciplinary team

## Abstract

**Introduction:**

Encephalocele is a herniation of intracranial contents through a defect in the skull bone. It is a distressing condition that poses significant technical challenge to the managing team, especially in a low-resource setting. In this report we present our experience of managing a case of Frontoethmoidal encephalocele in a 4-month-old infant.

**Case presentation:**

A 4-month-old infant was referred to our center with progressively enlarging mass over the dorsum of the nose. A diagnosis of frontoethmoidal encephalocele and hydrocephalus was established and subsequently, a multidisciplinary team was formulated for the management. An autologous calvarial bone graft was utilized to reconstruct the defect and the clinical outcomes were satisfactory given the circumstances.

**Discussion:**

Frontoethmoidal encephalocele is infrequently encountered in our experience. It poses a technical challenge for reconstruction. Satisfactory outcomes can be obtained by multidisciplinary team approach. The overall goal of the surgery is the reduction of healthy brain tissue, resection of dysplastic tissue and sac, watertight durable dural closure (either primary or with pericranium flap) and reconstruction of skull defect, using either autologous calvarial bone graft or prosthetic materials (such as titanium mesh, or bone filler).

**Conclusion:**

Despite its rarity and the technical challenges it poses, frontoethmoidal encephalocele can be successfully managed by a multidisciplinary team in a low-resource setting, even in the absence of prosthetic materials.

## Highlights

• This case report presents the successful surgical management of a frontoethmoidal encephalocele in a 4-month-old infant.
• We highlight the challenges and innovative strategies employed in a low-resource setting.

## Abbreviations

CSF: Cerebro Spinal Fluid
VPS: Ventriculoperitoneal shunt
ICP: Intracrainal pressure
NTD: Neural Tube defect

## 1 Introduction

Encephalocele is a herniation of the meninges, with or without the brain or the ventricles, through a defect in the skull bone. This congenital defect poses a significant danger to the infant, distress to the parents, and a surgical challenge to the managing team.

In this report, we present our experience of managing a case of Frontoethmoidal encephalocele in a low-resource setting. We elaborate on the unique challenges and surgical techniques used to address them. This case has been reported in line with the SCARE criteria [1].

## 2 Case presentation

**Fig. 1:**
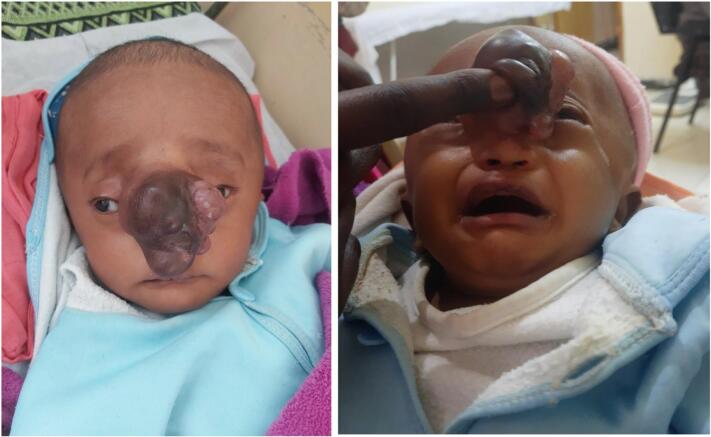
Initial picture showing large mass over the dorsum of the nose.

A 4-month-old firstborn male infant was referred to our center with progressively enlarging mass over the dorsum of the nose since birth. The mass was initially small but progressively enlarged over several weeks. There was also darkening of the skin overlying the mass (Fig. 1). The patient experienced progressive enlargement of his head size and had symptoms of snoring and frequent awakening from sleep. There was also increased distance between the eyes. Otherwise, there was no weakness, difficulty of suckling, or any abnormal body movement. The mother had been on antenatal care follow-up with folic acid supplementation based on the national guideline.

On physical examination vital signs were normal. The head circumference was 49 cm, which was >2SD/97th percentile for his age. There was an 8 × 7 × 6 cm tense multiloculated mass with cystic consistency over the nasal bridge area. Transillumination of the mass was possible using a torch.

**Fig. 2:**
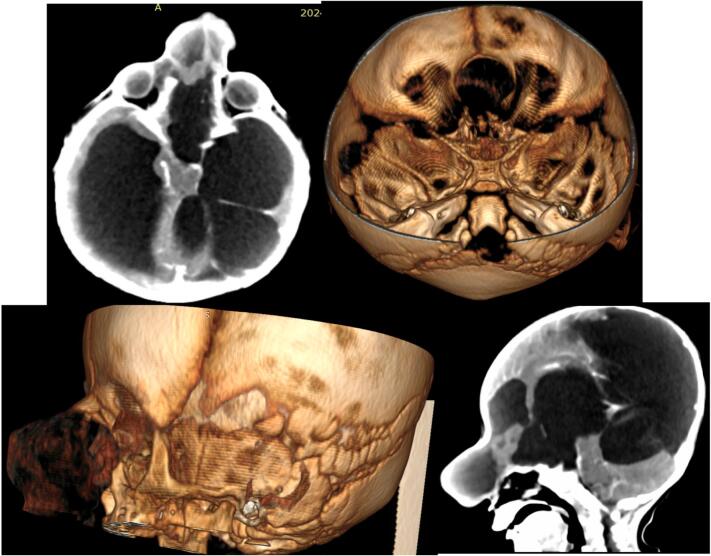
CT scan with 3D reconstruction showing the bone defect, protrusion of intracranial contents and hydrocephalus.

With clinical diagnosis of encephalocele and hydrocephalus CT scan of the head was obtained. The Imaging showed protrusion of part of the brain and CSF through a bone defect over the frontal-ethmoidal area that displaced the nasal bone (Fig. 2). There was significant enlargement of the ventricles with abnormal ventricular cyst in the left lateral ventricle.

Since such an anomaly was a difficult encounter in our setup, a multidisciplinary team was constituted involving neurosurgery & plastic surgery teams. A surgical management plan was then laid out taking into account the current capability of our center.

**Fig. 3:**
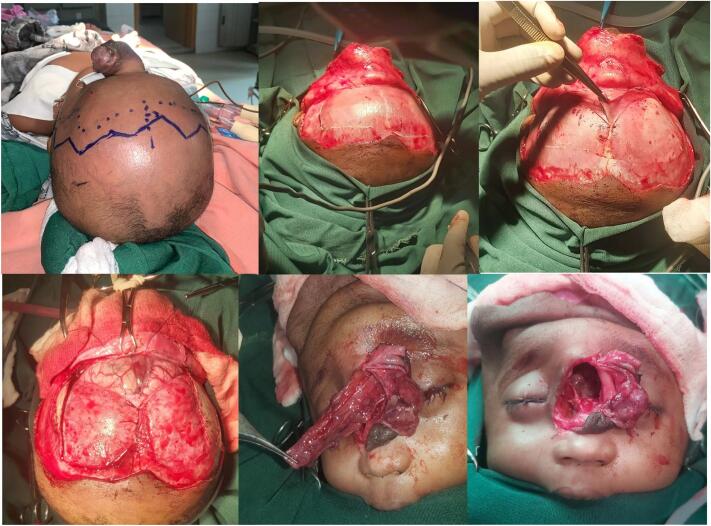
Intraoperative pictures demonstrating the steps followed during the procedure.

The patient was then prepared for surgery under general anesthesia in a supine position. Diluted Lidocaine with adrenalin 1:100,000 was infiltrated to the incision site. A formal coronal incision of the scalp was made extending from the tragus on each side (Fig. 3). Epicranium of the frontal bone was carefully raised based anteriorly for later on use as dural closure (Fig. 3). Bifrontal craniotomy was then made. To obtain adequate working space, ∼30 cc of CSF fluid was then drained from the lateral ventricle with a 10 cc syringe. This led to slackening of the tense intracranial structures which sagged the frontal lobe for better exposure of the defect site.

Dura was then opened. At this stage elliptical incision was made over the mass over the dorsum of the nose. After adequate and meticulous dissection through the nasal incision, intradural reduction of the herniated normal brain tissue was possible. Excision of the abnormal dysplastic brain and fibrous tissue was then performed. A 2 × 2 cm frontoethmoidal bone defect was then clearly encountered (Fig. 3).

The bone defect was reconstructed with a calvarial bone graft taken from the posterior edge of the harvested frontal bone on the margin forming the anterior fontanel. Titanium mesh was not used in this case as it was unavailable in our setup.

**Fig. 4:**
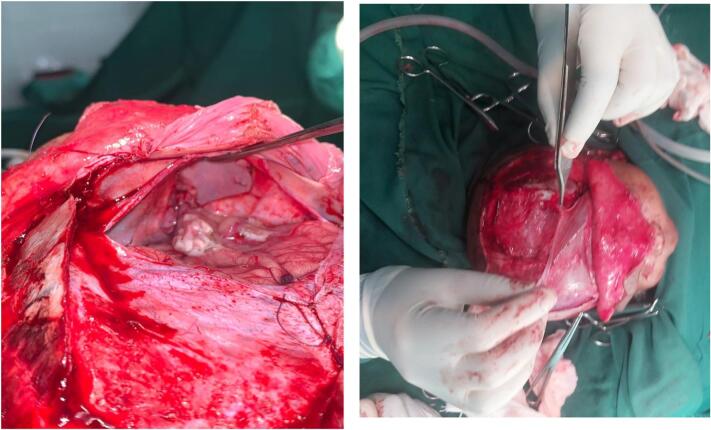
Picture showing an inlay bone graft used to reconstruct the bone defect and epicranial flap that was used to reinforce the closure site.

The bone graft was very thin to support a plate and screw for fixation, so Prolene 3-0 sutures were used to fix it over the defect as an inlay manner (Fig. 4). The epicranium from the frontal bone was then turned over and used to reinforce the bone graft. At this stage thorough irrigation of the intracranial structures were made using normal saline and the dura was closed in a water tight manner with Polyglycolic acid 4-0.

The craniotomy bone was placed back, fixed with few absorbable stiches and scalp wound was closed.

The pseudo hypertelorism was corrected through the nasal incision by placing inter-fixing suture between the two medial canthal ligaments using Prolene 3.0. A small remaining bone from the frontal bone was used to augment the nasal bridge and the excess discolored skin was then excised. The nasal incision was closed primarily.

**Fig. 5:**
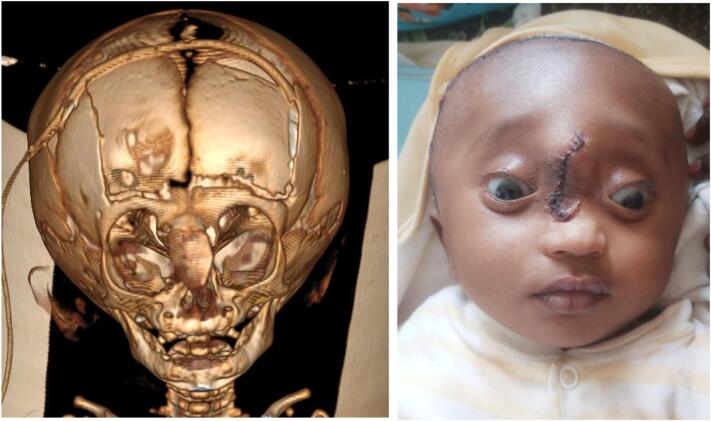
Postoperative follow-up CT scan and picture demonstrating good bone reconstruction and better aesthetic outcome.

Postoperatively the patient was put on prophylactic antibiotics. He was followed for 7 days and there was no major complication. He was finally discharged with follow-up visit instructions. The patient's parents were satisfied with the surgical outcome (Fig. 5).

## 3 Discussion

Encephalocele is a herniation of the meninges with or without the brain or the ventricles through a defect in the skull bone [2]. It is mainly a congenital entity believed to occur as part of neural tube defect. Some express skepticism about it being part of NTD and imply environmental and genetic factors as its cause [[3], [4], [5]]. The rationale behind this is that encountering such cases in babies born to mothers who received ideal antenatal care is not uncommon, as demonstrated in our case. There was also no clear predisposing risk factor identifies in our case. Rarely encephalocele can be an acquired condition from trauma, tumor or iatrogenic injury [6].

Encephalocele can be classified based on the location of the defect (see Table 1). The occipital area is the commonest location (75–90 %), followed by Frontoethmoidal location (13–15 %) [2,7,8]. The exact anatomic site of defect in sincipital encephalocele is the junction between frontal and ethmoidal bone at the foramen cecum [2]. Our patients encephalocele that displaced the nasal bone seems to fall under Naso-ethmoidal type of frontoethmoidal encephalocele (see Table 1).

**Table 1:** Suwanwela and Suwanwela anatomic classification of encephalocele (1972) [9].

Location	Classification	Sub divisions
Occipital		
Sincipital	Frontoethmoidal	1. Nasofrontal – herniation between the frontal & nasal bones and appears at the root of the nose, above the level of the nasal bone (fonticulus frontalis). 2. Naso-ethmoidal – herniation between the nasal bones and nasal cartilage and is located inferior to the nasal bones 3. Naso-orbital – Herniation through a defect in the maxillary frontal process and causes proptosis and displacement of the globe
	Inter frontal	
	Associated with craniofacial clefts	
Convexity		1. Inter-frontal 2. Anterior fontanel 3. Inter parietal 4. Posterior fontanel 5. Temporal
Basal		1. Trans-ethmoidal 2. Sphenoethmoidal 3. Trans-sphenoidal 4. Fronto sphenoidal or spheno-orbital

The incidence of Frontoethmoidal encephalocele is not well studied in Ethiopia, but studies from other countries indicate it to be a rare condition. The overall incidence is 0.8–4 cases per 10,000 live births [4,8]. Frontoethmoidal encephalocele is also infrequently encountered in our experience.

Localizing the exact site of the defect needs extensive evaluation with modern imaging modalities including MRI which is a gold standard, or CT scan [4,7,10]. MR angiography can also be utilized and has the advantage of evaluating for the presence of major vessels in the encephalocele and assessing relation with the dural sinus [4,7].

In addition to identifying the site of the defect, imaging will reveal additional brain anomalies, such as hydrocephalus and ventricular cyst which are crucial for surgical planning [2,10].

Surgical correction is the sole management modality upon the confirming the diagnosis [10,11]. These anomalies should be repaired in the first few months of life. Early repair prevents rupture and decreases the risk of CNS infection, neurological deficits, facial disfigurement and vision impairment [2].

Unrepaired Frontoethmoidal encephaloceles may lead to significant distortion of facial anatomy during growth mandating early repair [2]. Operation is ideally made after 4 months and should always be weighed against the risk of significant blood loss and anesthesia risks such as hypothermia [2,7,10].

Management requires a multidisciplinary team of surgeons, involving neurosurgery, maxillofacial and plastic surgery, working in harmony [[2], [3], [4]]. There are several techniques for repair of frontoethmoidal encephaloceles. This include the classic Tessier approach, the Chula technique & modified Chula techniques. However no single technique has been established as superior to others so far [3]. The main difference between our technique and the Chula technique is the utilization of bone that is harvested from the posterior edge of the frontal bone. In addition, rather than costochondral graft for nasal augmentation remaining skull bone graft was used. This was technically easy to achieve.

The goal of the surgery is the reduction of healthy brain tissue, resection of dysplastic tissue and sac, watertight durable dural closure (either primary or with pericranium flap) and reconstruction of skull defect, using either autologous calvarial bone graft or prosthetic materials (such as titanium mesh, or bone filler) [2,3].

In selected cases, placement of a VPS might be needed prior to any attempt at reconstructing the encephalocele [11]. In our case needle drainage sufficed to mitigate the hydrocephalus. Drainage of CSF should be executed carefully to avoid complications such as hypotension, subdural hematomas, or arrhythmias [2].

Encephalocele poses a significant reconstructive challenge especially in a low-resource setting. The unavailability of titanium mesh makes the standard repair difficult and impossible at times. In such scenarios, the use of autologous sources might be the only available option.

The long-term outcome of patients undergoing autologous bone graft reconstruction needs a comparative study with those who had a prosthetic implant to establish a clear management guideline.

Postoperative follow-up of patients is critical to recognize and intervene for possible complications such as CSF leak, meningitis, graft displacement and raised ICP [2,10,11]. This complication can be avoided by careful preoperative planning, meticulous surgical techniques, and multidisciplinary team management [11].

Aesthetic outcomes are generally excellent. In a case series study the aesthetic outcomes were excellent in 70 %, satisfactory in 18 %, and poor in only 3 % of cases [2]. Few patients might require future revision rhinoplasty depending on the need as an adult [2].

In the absence of gross brain injury or herniation, patient is expected to have normal growth, intelligence and motor development [2]. When compared, frontoethmoidal location has a significantly better prognosis (100 %) than occipital location (55 %) [10]. The skull defect is also expected to heal well due to the healing potential of infants and remodeling of the bone with growth [3,7].

## 4 Conclusion

In conclusion, encephalocele poses a significant challenge in resource-limited setups. The bone defect might need a prosthetic material for definitive reconstruction, but in cases where these options are not available, a calvarial bone graft is a suitable alternative. A multidisciplinary team approach should always be utilized for good patient outcomes.

We wanted to report this case to share our experience in managing such cases in low-resource settings and provide readers with a comparative case for future encounters in similar setups.

## Ethical approval

Ethical approval or clearance was not required for a case report publication from our institution (IRB of Addis Ababa University, College of Health Sciences).

## Funding

This case report did not receive any form of funding or grant from public, private, or non-profit organizations.

## Author contribution

Dr. Anteneh Meaza Dawit: Drafting, writing of manuscript and management of the patient

Dr. Getaw Alamne Gebeyehu: Revising, approving of the manuscript and management of the patient

Dr. Salahadin Bedru Mohammed: Approving the manuscript and management of the patient

## Guarantor

Dr. Anteneh Meaza Dawit will take full responsibility for the work.

## Consent

Written informed consent was obtained from the patient's parents for publication of this case report and accompanying images. A copy of the written consent is available for review by the Editor-in-Chief of this journal upon request.

## Conflict of interest statement

No conflict of interest has affected this report.
